# Platelet-activating factor receptor (PAF-R)-dependent pathways control tumour growth and tumour response to chemotherapy

**DOI:** 10.1186/1471-2407-10-200

**Published:** 2010-05-13

**Authors:** Soraya I de Oliveira, Luciana NS Andrade, Ana C Onuchic, Sueli Nonogaki, Patrícia D Fernandes, Mônica C Pinheiro, Ciro BS Rohde, Roger Chammas, Sonia Jancar

**Affiliations:** 1 Department of Immunology, Institute of Biomedical Sciences, University of Sao Paulo, Sao Paulo, Brazil; 2 Department of Radiology, University of Sao Paulo Medical School, University of Sao Paulo, Sao Paulo, Brazil; 3 Department of Pharmacology, Institute of Biomedical Sciences, Federal University of Rio de Janeiro, Rio de Janeiro, Brazil; 4 Adolfo Lutz Institute, Sao Paulo, Brazil

## Abstract

**Background:**

Phagocytosis of apoptotic cells by macrophages induces a suppressor phenotype. Previous data from our group suggested that this occurs via Platelet-activating factor receptor (PAF-R)-mediated pathways. In the present study, we investigated the impact of apoptotic cell inoculation or induction by a chemotherapeutic agent (dacarbazine, DTIC) on tumour growth, microenvironmental parameters and survival, and the effect of treatment with a PAF-R antagonist (WEB2170). These studies were performed in murine tumours: Ehrlich Ascitis Tumour (EAT) and B16F10 melanoma.

**Methods:**

Tumour growth was assessed by direct counting of EAT cells in the ascitis or by measuring the volume of the solid tumour. Parameters of the tumour microenvironment, such as the frequency of cells expressing cyclo-oxygenase-2 (COX-2), caspase-3 and galectin-3, and microvascular density, were determined by immunohistochemistry. Levels of vascular endothelium growth factor (VEGF) and prostaglandin E2 (PGE2) were determined by ELISA, and levels of nitric oxide (NO) by Griess reaction. PAF-R expression was analysed by immunohistochemistry and flow cytometry.

**Results:**

Inoculation of apoptotic cells before EAT implantation stimulated tumour growth. This effect was reversed by *in vivo *pre-treatment with WEB2170. This treatment also reduced tumour growth and modified the microenvironment by reducing PGE2, VEGF and NO production. In B16F10 melanoma, WEB2170 alone or in association with DTIC significantly reduced tumour volume. Survival of the tumour-bearing mice was not affected by WEB2170 treatment but was significantly improved by the combination of DTIC with WEB2170. Tumour microenvironment elements were among the targets of the combination therapy since the relative frequency of COX-2 and galectin-3 positive cells and the microvascular density within the tumour mass were significantly reduced by treatment with WEB2170 or DTIC alone or in combination. Antibodies to PAF-R stained the cells from inside the tumour, but not the tumour cells grown *in vitro*. At the tissue level, a few cells (probably macrophages) stained positively with antibodies to PAF-R.

**Conclusions:**

We suggest that PAF-R-dependent pathways are activated during experimental tumour growth, modifying the microenvironment and the phenotype of the tumour macrophages in such a way as to favour tumour growth. Combination therapy with a PAF-R antagonist and a chemotherapeutic drug may represent a new and promising strategy for the treatment of some tumours.

## Background

There is evidence that macrophages have the capacity to change their phenotype in response to changes in the microenvironment. It has been suggested that M1 and M2 represent the extremes of a variety of phenotypes that macrophages can express and that the M2 phenotype is associated with suppressor functions [1,2]. It has been shown that apoptotic cells induce macrophage polarization towards a suppressive phenotype. Fadok et al [3] reported that the addition of apoptotic cells to LPS-stimulated macrophages shifts the type of mediators/cytokines produced from a pro-inflammatory towards a suppressive profile. The recognition of apoptotic cells by macrophages is achieved through molecules that are expressed in the plasma membrane of apoptotic cells and bind to a variety of receptors present on the surface of the macrophages, resulting in the phagocytic removal of altered or dying cells [4]. We have previously found that the rate of phagocytosis of apoptotic cells is higher than that of viable cells and that this potentiation is abolished if macrophages are treated with an antagonist of the PAF-R. Moreover, the interaction of macrophages with apoptotic cells induces the expression of COX-2, the inducible enzyme that is responsible for the synthesis of prostaglandins and is also inhibited by treatment with the PAF-R antagonist [5]. One of the products of this enzyme is PGE2, which exerts suppressive actions through interaction with EP2 or EP4 receptors in the macrophage [6]. PAF-R is a G-protein-coupled receptor that is present in the plasmatic and nuclear membrane and also in the cytoplasm of various cell types including macrophages. Depending on its localization, the receptor is linked to different sub-units of G-protein, Gα_q _or Gα_i/o _and thus activates distinct intracellular signaling cascades [7].

The findings that apoptotic cells share common ligands with PAF, that apoptotic cells dampen macrophage activation and that PAF-R is somehow involved in these effects could be particularly relevant in the case of tumour growth. As the number of tumour cells increases during tumour development, many of these cells die by apoptosis or necrosis due to the reduction in oxygen and nutrient supply. Induction of apoptosis is also the mechanism of action of anti-tumour chemotherapy. Correa et al [8] clearly demonstrated that apoptotic cells injected together with a sub-tumourigenic dose of B16F10 melanoma cells promote tumour growth. This could be attributed to the postulated suppressor effect of apoptotic cells on macrophages but whether it is dependent on PAF-R remains to be determined. Antagonists of PAF-R have been tested in some tumours: in human breast cancer they inhibited cell proliferation *in vitro *and reduced the formation of new vessels in tumours induced by implantation of these cells [9]; in B16F10 murine melanoma they decreased lung metastasis [10]; in EAT they reduced tumour growth [11].

Based on the data discussed above we postulated that the interaction of macrophages with apoptotic cells in the tumour microenvironment, through PAF-R-dependent mechanisms, would drive macrophage polarization towards a suppressive phenotype favoring tumour growth. In the present study we investigated this hypothesis in two murine tumours: EAT and melanoma B16F10. Apoptotic cells were either inoculated into the tumour or induced by dacarbazine (DTIC), an agent that is widely used in human melanoma therapy. The role of the PAF-R was evaluated using the antagonist WEB2170. Tumour growth was evaluated by counting the cells in the ascitis or measuring tumour volume. The tumour microenvironment elements that were analysed were the levels of PGE2, VEGF and NO in the ascitis and, in the melanoma, the number of cells expressing COX-2, the number of intra-tumoural blood vessels and tumour infiltration by activated macrophages/dendritic cells expressing galectin-3, which is associated with the suppressive M2 phenotype. PAF-R expression was evaluated in cells from inside the tumour and in melanoma cells taken from cultures.

## Methods

### Animals

Seven- to 10-week-old male BALB/C and 7- to 8-week-old female C57BL/6 mice from our own animal facilities were used. All animal procedures were in accordance with the ethical principles adopted by the Brazilian College of Animal Experimentation and approved by the Ethical Committee for Animal Research of the Institute of Biomedical Sciences, University of São Paulo.

### Tumours

The murine melanoma cell line B16F10 was a kind gift from I. Fidler (M.D. Anderson, Texas, USA) and was cultured in RPMI 1640 medium (Cultilab, Campinas, Brazil), pH 7.4 supplemented with 10% fetal bovine serum (FBS, Cultilab, Campinas, Brazil), in the absence of antibiotics, at 37°C and 5% CO_2_. Ehrlich ascitis tumour (EAT) cells were grown as described elsewhere [12].

### Evaluation of tumour growth

Ehrlich ascitis tumour (10^3^-10^5 ^cells) was injected intraperitoneally (i.p.) in BALB/c mice. At different times after tumour implantation, aliquots of the ascitis fluid were recovered using a 21-gauge needle to measure cell number and mediator/cytokine levels. B16F10 melanoma cells (5 × 10^5^) were injected subcutaneously (s.c.) in C57BL/6 mice and tumour growth was determined via measurement of the diameter of the solid tumour mass, from which the volume was estimated using the formula for a spheroid: V = 0.52 × (largest axis) × (smallest axis) ^2^.

### Treatments

The PAF-R antagonist WEB2170 (5 mg/Kg; Boehringer Ingelheim, Germany) was injected i.p. 30 minutes before tumour implantation and injections were repeated every 24 h until the end of the experiment. In order to evaluate the possible benefit of the combined therapy with WEB2170, treatment with the chemotherapeutic drug DTIC was chosen at a suboptimal regimen based on previously published studies [13,14]. In our study 40 μg DTIC (Sigma Chem. Co.) was given i.p. to each mouse every 3 days after tumour implantation. To determine the impact of these treatments on the survival of B16F10 melanoma-bearing mice, the treatment protocols were extended to 35 days. The Kaplan-Meier method and the log-rank test were used to estimate and compare the different groups.

### Quantification of VEGF, PGE2 and nitrite in the ascitis fluid

At day 10 of EAT growth, BALB/C mice were sacrificed and the ascitis fluid was recovered. The suspension was centrifuged (180 × *g *for 10 min) and VEGF levels were determined in the supernatant by ELISA using goat polyclonal antiserum against mouse VEGF (Santa Cruz Biotechnology, Inc.). PGE2 levels were also determined by ELISA using a commercially available kit (Cayman Chem. Co.). NO levels were determined according to the method described by Bartholomew (1984) [15]. Briefly, aliquots of 150 μL of the ascitis fluid were treated with 15 μL of 10% ZnSO_4 _solution for 10 minutes at 4°C. Three μL of 2.5 N NaOH were added and the ascitis fluid was incubated at 4°C for 10 minutes. The NO_3_^- ^content was converted to NO_2_^- ^through addition of 15 μL of 0.5 M NaH_2_PO_4_, 15 μL of 2.4 M ammonium formiate and 10 μL of a suspension of *E. coli *to the ascitis fluid. The bacterium *E. coli *expresses nitrate reductase, which catalyses the conversion of nitrate to nitrite. Samples were further incubated at 37°C for 2 h, centrifuged (18000 × *g *for 5 min at 20°C), and 100 μL of Griess Reagent (0.1% N-naphthyl-ethyl-indiamine, 1% sulphanilamide, 2.5% orthophosphoric acid - Sigma Co.) were added to the same volume of the supernatant. NO levels were quantified in a spectrophotometer at 540 nm.

### Characterization of apoptotic and necrotic cells in the ascitis fluid

BALB/C mice were inoculated i.p. with 1 × 10^3 ^EAT cells and sacrificed after 7 or 10 days. The peritoneal cavity was injected with 3 mL of PBS to recover the ascitis. The fluid was centrifuged (180 × *g *for 10 min) and the cell pellet resuspended in 1 ml of RPMI 1640 with 10% fetal calf serum. Trypan Blue dye (0.2%) was added to aliquots of cellular suspensions and the percentage of cells that had taken up the dye (necrotic cells) was determined microscopically. The percentage of apoptotic cells was determined by flow cytometry (FACScalibur, Becton Dickinson, San Jose, CA) after incubation of 3 to 5 × 10^5 ^cells with propidium iodide (20 μg/mL in 0.1% sodium citrate and 0.1% Triton X-100) at 4°C for 2 h in the dark.

### Induction of apoptosis in murine thymocytes

Murine thymocytes were obtained through surgical extraction of the thymus from BALB/C mice. After thymus dissociation, the thymocyte suspension was centrifuged (400 × *g*, 4°C, 7 min), the pellet was resuspended in RPMI medium and cell viability was determined using Trypan Blue vital dye. After this, thymocytes were γ-irradiated (450 rad) and incubated at 37°C for 18 hours. Apoptotic cells were detected by annexin V staining and the percentage of apoptotic cells was measured by flow cytometry (FACS, Becton-Dickinson, CA), as described elsewhere [5]. To evaluate the effect of apoptotic thymocytes on EAT growth, BALB/C mice were inoculated with 6 × 10^6 ^viable or apoptotic thymocytes into the peritoneal cavity 2 h before the inoculation of 1 × 10^5 ^EAT cells. Animals were sacrificed after 5 days and aliquots of the ascitis fluid were recovered to estimate EAT growth via direct cell counting in a Neubauer chamber under a light microscope.

### Histological, immunohistochemistry and morphometric analysis of melanoma specimens

Tumours derived from B16F10 melanoma cells were excised and processed for immunohistochemistry to detect the expression of galectin-3 (1:32, ATCC), PAF-R (1:80; Cayman), COX-2 (1:100; Santa Cruz) and activated caspase-3 (1:600; Cell Signaling). Reactions were performed as described elsewhere [16,17]. Images of different areas of each tumour specimen were acquired with a digital camera DXM 1200F (Nikon) and quantitative analysis was performed using Eclipse Net software (Nikon). For percent area measurements, grids were projected on tissue sections and the number of grid intersections that overlaid a cell with a specific type (identified by immunohistochemistry reaction) or functional blood vessels (identified by routine H&E staining and by the identification of blood cells) was counted. Data were expressed as percent area occupied by vessels or cells expressing a given marker (COX-2-expressing cells, galectin-3-expressing cells, and activated caspase-3-expressing cells). The total number of cells positively stained by each marker was determined by counting multiple grids randomly placed on tissue sections at medium power field magnification (40×). Spleens were also processed for immunohistochemistry to detect cells expressing galectin-3 in the white pulp.

### Preparation of cell suspensions from tumours

Tumours were excised and kept in RPMI 1640 at 4°C for immediate processing. All collected tumours were washed in ice-cold PBS, finely minced and transferred to 60 mm-diameter Petri dishes containing 1 mL MTH (HBSS, 0.5 U/mL DNAse in 15 mM HEPES) supplemented with 0.15 Wunsch units/mL of Liberase Blendzyme 3 (Roche). Fragments were incubated for 30 minutes at 37°C under constant agitation (80 rpm) for mechanical dissociation. Cell suspensions were then filtered using nylon membranes (41 μm pores). The filtrate was centrifuged at 4°C (1200 rpm for 5 min), washed in cold PBS and resuspended in 3 mL of red blood cell lysis solution (Amersham Biotech). After 3 min, another 3 mL of RPMI 1640 was added to the suspension, which was then centrifuged as above and washed in PBS. Cell number was then determined by direct counting using a Neubauer chamber.

### Flow cytometry

For flow cytometry experiments, a million cells were separated for each reaction tube or well. Cells were washed with PBS-BSA (PBS containing 0.5% BSA) and then incubated with an Fc blocker antibody for 15 min at 4°C. Cells were then centrifuged (1200 rpm for 5 min at 4°C), washed with PBS-BSA and incubated with 0.3 μg/mL of a rabbit polyclonal anti-PAF-R antibody (Cayman Chemical, USA) for 60 min on ice. After washing, cells were incubated with a FITC-labeled anti-rabbit IgG for 30 min on ice in the dark. After washing, the cells were then suspended in 2% formaldehyde in PBS and kept in the dark until acquisition and analysis in a flow cytometer (FACScalibur, Becton Dickinson, San Jose, CA, USA). Histogram analysis was done using the CellQuest software. The control samples were incubated with the Fc blocker and with the FITC-labeled secondary antibody.

### Statistical analysis

Data were analysed using ANOVA followed by Tukey or Bonferroni post-hoc tests. Survival was analysed by the Kaplan-Meier method and survival curves were compared using the log-rank test. Differences were considered significant when p < 0.05.

## Results

### Apoptotic cells favour EAT growth through PAF-R-dependent pathways

**Figure 1:**
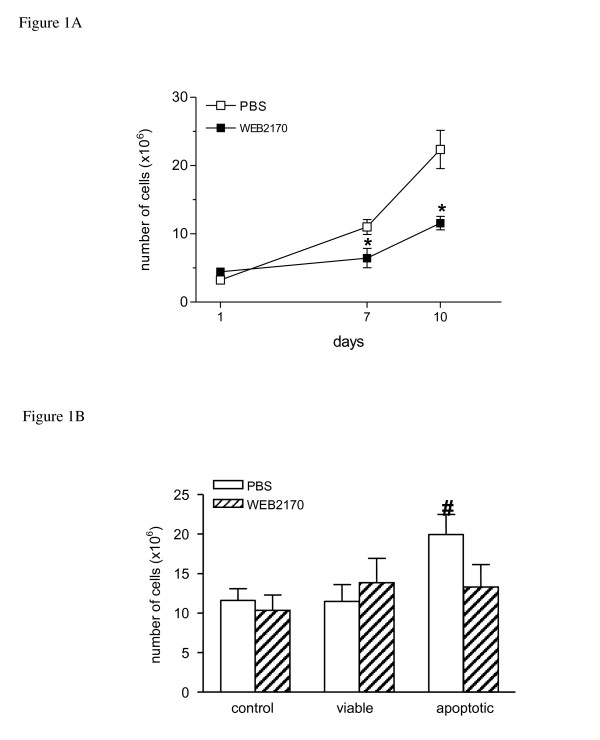
**Phagocytosis of apoptotic cells through PAF-R affects EAT growth**. EAT cells (1 × 10^3 ^cells) were inoculated into the peritoneal cavity of BALB/c mice. Ascitis fluid was collected at different times after tumour implantation and the cell number was determined using a Neubauer chamber. **(A) **Animals were injected i.p. with phosphate buffered saline (PBS) or WEB2170 (5 mg/Kg) 30 min before EAT cells and daily for 6 days (n = 3-4). **(B) **Live or apoptotic thymocytes (6 × 10^6^) were inoculated i.p. 2 h before implantation of EAT. WEB2170 (5 mg/Kg) was given i.p. 30 min before the thymocytes. Cell number was determined 5 days after tumour implantation (n = 17-21). Data represent the mean ± SEM of the number of EAT cells. Statistical analyses were performed using ANOVA and SNK (Student Neumans-Keuls test) and differences were considered significant at p < 0.05. (*) p < 0.05 compared to control groups.

Fig. 1A shows the kinetics of EAT growth following implantation of 1 × 10^3 ^tumour cells into the peritoneal cavity of BALB/c mice. The number of cells present in the ascitis fluid was counted on days 1, 7 and 10 after implantation. It can be seen that the number of cells in the peritoneal cavity increased progressively. Whereas at day 7, around 92% of the cells were EAT cells, at day 10 virtually all cells identified in the differential counts were EAT cells. Treatment with the PAF-receptor antagonist WEB2170 (5 mg/kg) significantly reduced the number of tumour cells, markedly after 10 days of treatment (Fig. 1 A). The percentage of apoptotic cells in the ascitis fluid after 7 and 10 days of tumour growth was less than 2%. The percentage of necrotic cells was also less than 2%, although this percentage was lower at 10 days compared to 7 days of tumour growth in the control PBS group (Table 1). Whereas in the non-treated group, the percentage of necrotic cells tended to decrease with time, in the treated group, the percentage of necrotic cells remained constant. On the other hand, considering the total number of cells in the peritoneal cavity at these time-points of tumour growth, the absolute quantities of both apoptotic and necrotic cells were higher in the animals within the non-treated group.

**Table 1:** Effect of WEB2170 treatment on the percentage of apoptotic and necrotic cells during EAT growth.

Apoptotic cells	% of total cells			
	
	PBS		WEB2170	
	
	Day 7	Day 10	Day7	Day 10
	0.98 ± 0.98	1.38 ± 0.62	0.62 ± 0.26	1.72 ± 0.45

**Necrotic cells**	1.50 ± 0.35	0.30 ± 0.11^(#)^	0.60 ± 0.31^(*)^	0.70 ± 0.30

1 × 10^3 ^EAT cells were i.p. injected in BALB/C mice and the ascitis fluid was collected after 7 and 10 days to determine the percentage of apoptotic and necrotic cells. WEB2170 (5 mg/Kg; i.p.) was given 30 min before inoculation of EAT cells.

(*) p < 0.05 comparing WEB2170-treated with PBS group

(#) p < 0.05 comparing day 10 with day 7 within PBS or WEB2170 group

n = 3-9 animals/group

In another set of experiments, Balb/c mice received an i.p. injection of 6 × 10^6 ^live or apoptotic thymocytes 2 h before implantation of EAT into the peritoneal cavity. Animals were sacrificed at day 5 of tumour growth and the total number of cells present in the ascitis fluid was determined (at that time more than 90% of the peritoneal cells were tumour cells). The results showed that previous inoculation of apoptotic but not live thymocytes increased EAT growth, as total cell counts were about 2-fold higher (*p *< 0.05) in this group than in the control group (Fig. 1B). Mice were also either treated with WEB2170 or PBS (as a control) 30 min before inoculation of the thymocytes to investigate the possible involvement of PAF-R in the potentiating effect of apoptotic cells. We observed that treatment with a single dose of WEB2170 reverted the stimulatory effect of apoptotic cells on EAT growth, whereas no effect was observed on tumour growth in mice inoculated with viable cells (Fig. 1B).

### Activation of PAF-R-dependent pathways led to increased secretion of PGE2, VEGF and NO in the ascitis fluid

**Figure 2:**
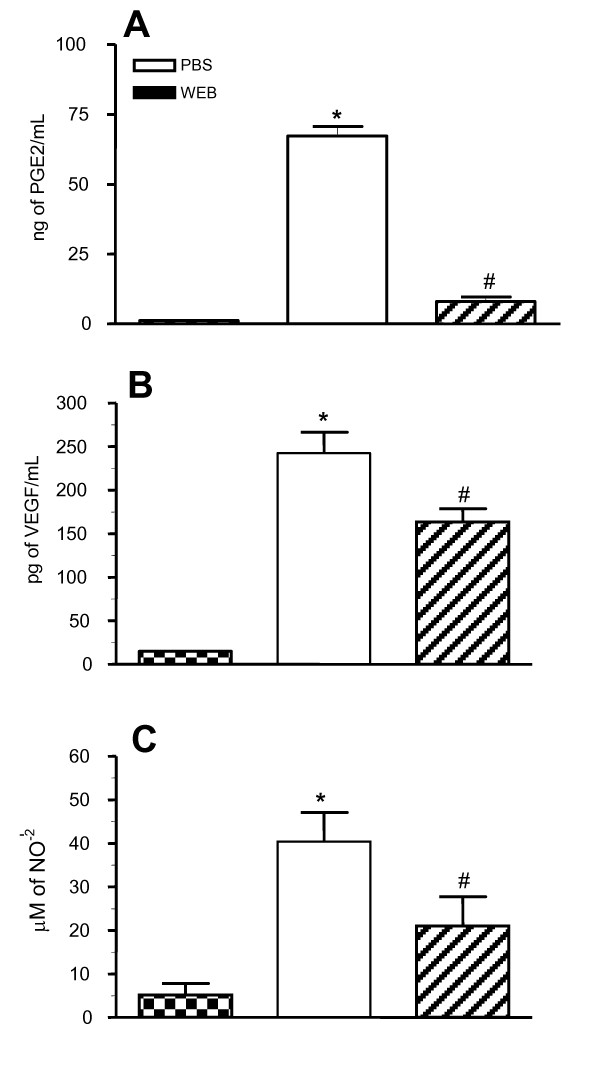
**PAF receptor antagonist reduces PGE2, VEGF and NO levels in the ascitis fluid**. EAT cells (1 × 10^5^) were inoculated i.p. into BALB/c mice and the levels of PGE2, VEGF and nitric oxide (NO) were measured in the ascitis fluid 7 days later. One group of mice was treated with WEB2170 (5 mg/Kg), 30 min before tumour implantation and daily up to the 6^th ^day. Data represent the mean ± SEM (n = 3-7). Statistical analyses were performed using ANOVA and SNK (Student Neumans-Keuls test) and differences were considered significant at p < 0.05. (*) p < 0.05 compared to non-treated group. In each graph, the first column represents the baseline levels, determined in the washings of the peritoneal cavity of mice without tumours, the second column represents the levels in tumour-bearing mice without treatment (PBS-control group), and the third column represents tumour-bearing mice treated with WEB2170.

High levels of PGE2, which has a suppressor effect on some macrophage functions [11], and of VEGF, which plays an important role in tumour growth due to its pro-angiogenic activity [18], were apparent in the mice peritoneal cavity during EAT growth. In addition, activation of PAF-R was shown to induce PGE2, VEGF and NO [19]. We then investigated the effect of a PAF-R antagonist on these molecules in the ascitis fluid 7 days after tumour implantation (1 × 10^5 ^EAT cells). Mice were treated with WEB2170, 30 min before tumour implantation and daily up to the 6^th ^day. The levels of PGE2 (Fig. 2A), VEGF (Fig. 2B) and NO detected as the NO_2_^- ^concentration (Fig. 2C) in the ascitis fluid were increased in EAT-bearing animals at this time. Treatment with WEB2170 significantly decreased the levels of all three mediators.

### Combined therapy with WEB2170 and DTIC in EAT growth

The pro-tumoural effect observed with the addition of apoptotic thymocytes before EAT implantation prompted us to evaluate a more realistic scenario, namely the impact of blocking PAF-R-dependent pathways during the course of chemotherapy. DTIC (40 μg injected i.p. every 3 days after tumour implantation) was chosen as the chemotherapeutic drug, based on its capacity to induce apoptosis in a wide variety of tumour cells in *in vivo *models [13]. It can be seen in Table 2 that the number of tumour cells 10 days after implantation in BALB/c mice inoculated i.p. with 1 × 10^5 ^EAT cells was significantly reduced by treatment with DTIC (77%) or WEB2170 (76%). The combined treatment with both DTIC and WEB2170 was around three times more effective than either treatment alone.

**Table 2:** Effect of WEB2170, DTIC and combined treatment on EAT growth.

Treatment	**×10**^**6 **^**cells**
**Non treated**	66.01 ± 16.07

**DTIC**	14.99 ± 6.16(*)

**WEB2170**	15.38 ± 4.26(*)

**W + D**	4.55 ± 2.16(*)

1 × 10^5 ^EAT cells were i.p. injected in BALB/c mice and after 10 days, the ascitis fluid was collected to determine tumour growth. WEB2170 (5 mg/Kg; i.p.) was given 30 min before inoculation of EAT cells and DTIC (40 μg/animal; i.p.) was given every 3 days after tumour implantation.

(*) p < 0.05 compared with non treated group

n = 5-9 animals/group

### Combined therapy with both WEB2170 and DTIC prolonged the survival of melanoma-bearing mice

**Figure 3:**
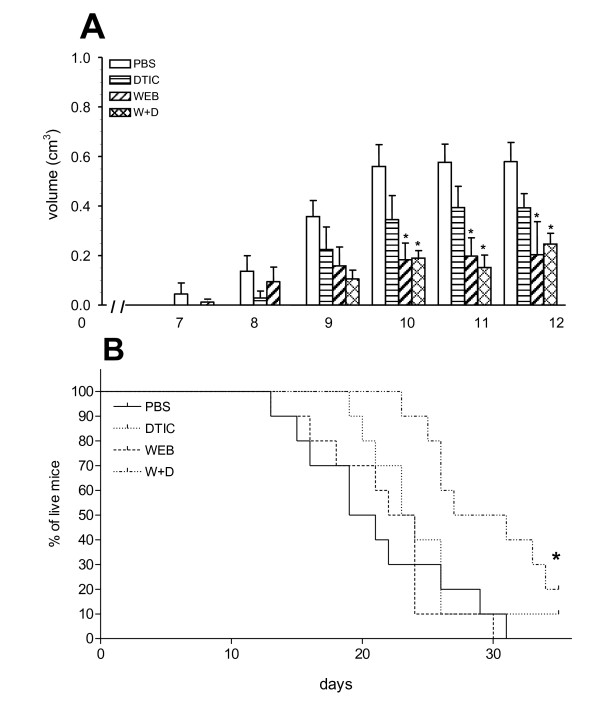
**PAF-receptor antagonist, WEB2170, inhibits melanoma growth and in combination with chemotherapy improves the survival of melanoma-bearing mice**. **(A) **B16F10 melanoma cells (5 × 10^5^) were injected s.c. into C57BL/6 mice and tumours were measured daily with a caliper. Tumour volume was calculated by the formula: maximum diameter × (minimum diameter)^2 ^× 0.52. WEB2170 (5 mg/Kg) was given i.p. 30 minutes before the tumour followed by daily injections for 12 days. DTIC (40 μg/animal) was injected i.p. every 3 days after tumour implantation. Data represent the mean ± SEM of tumour volume (n = 5). **(B) **The Kaplan-Mayer survival curve. For the survival experiments, WEB2170-treatment was given once a day and DTIC every 3 days for 35 days or until the animals died (n = 8-9). Statistical analyses were performed using the log rank test and differences were considered significant at p < 0.05. (*) p < 0.05 compared to PBS group.

The effect of treatments with WEB2170 and DTIC was then evaluated in a solid tumour, the B16F10 murine melanoma model. Tumour cells (5 × 10^5^) were injected s.c. in C57Bl/6 mice. Fig. 3A shows the kinetics of melanoma growth after tumour cell implantation. A marked delay in tumour growth was observed in animals receiving WEB2170, either alone or combined with DTIC, which was significant 10-12 days after tumour implantation (Fig. 3A). At day 12, animals were sacrificed and the tumours were analysed by immunohistochemistry. Regardless of the initial delay in tumour growth, survival of melanoma-bearing animals receiving WEB2170 alone was not significantly different from the survival of either melanoma-bearing animals in the control group (PBS group, Fig. 3B), or animals receiving DTIC alone. However, the association of both DTIC and WEB2170 prolonged the survival of melanoma-bearing mice significantly (Fig. 3B).

### Tumour microenvironment elements are targets for the combined therapy with DTIC and WEB2170 in murine melanoma

**Figure 4:**
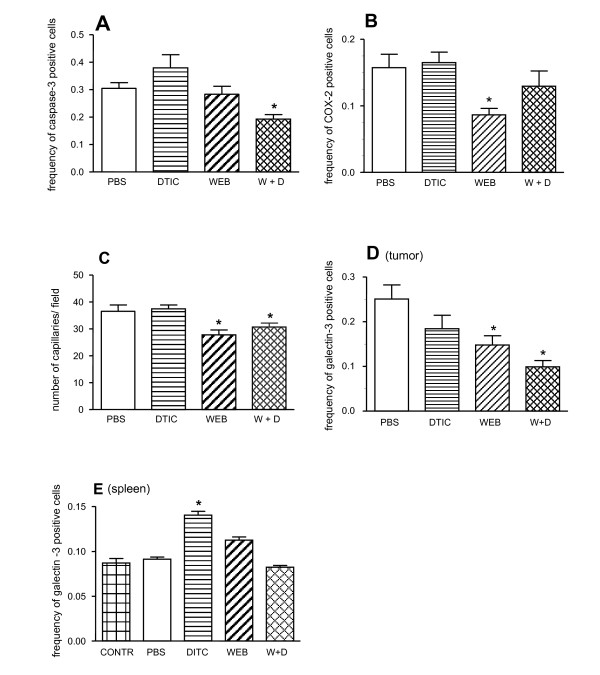
**Combined therapy with PAF-receptor antagonist and dacarbazine targets both tumour and microenvironmental elements**. B16F10 melanoma cells (5 × 10^5^) were injected s.c. into C57BL/6 mice and after 12 days the tumours were excised and processed for immunohistochemistry analysis with antibodies to caspase-3 **(A)**, COX-2 **(B)**, CD34 **(C) **or galectin-3 **(D)**. Alternatively, spleens of animals of the very same groups and of naïve mice were analysed for the presence of galectin-3-expressing cells in the white pulp **(E)**. WEB2170 (5 mg/Kg) was given 30 min before the tumour followed by daily injections for 12 days. DTIC (40 μg/animal) was injected i.p. every 3 days after tumour implantation. Morphometric analyses were performed using Eclipse Net software (Nikon). Grids were projected onto tissue sections and the number of grid intersections that overlaid an immunoreactive cell was counted. Results are expressed as the % of area occupied by the cells expressing a given marker (frequency). Data represent the mean ± SEM of positive cells (n = 10). Statistical analyses were performed using ANOVA and SNK (Student Neumans-Keuls test) and differences were considered significant at p < 0.05. (*) p < 0.05 comparing treated with non-treated (PBS) groups.

Tumour cell and tumour microenvironmental parameters, such as the relative frequency of apoptotic cells (caspase-3 positive) within the tumours (Fig. 4A), the relative frequency of COX-2 positive cells (Fig. 4B), microvascular density (Fig. 4C), and the relative frequency of galectin-3 positive cells, used as a marker for alternatively activated M2 macrophages or dendritic cells (Fig. 4D), were analysed via immunohistochemistry of tumours obtained from the four groups studied, namely control (PBS group), DTIC or WEB2170 (DTIC or WEB groups), and combined DTIC and WEB2170 (W + D group).

Apoptotic cells were quantified using antibodies reacting with activated caspase-3. The frequency of caspase-3 positive cells within the tumour was slightly but significantly lower in the group receiving both DTIC and WEB2170. No significant changes were found in the other groups (Fig. 4A). Although statistically significant, this slight decrease in the frequency of apoptotic cells within the tumours of animals treated with both DTIC and WEB2170 may not be the cause of the prolonged survival of this group of animals. Regardless, the result suggests that prolonged survival was not due to a direct increase in the cytotoxic effect of DTIC on tumour cells. If this were the case, we would expect to observe an increase in apoptotic cells within the tumour. We then investigated whether the treatments described above interfered with selected aspects of the tumour microenvironment.

**Figure 5:**
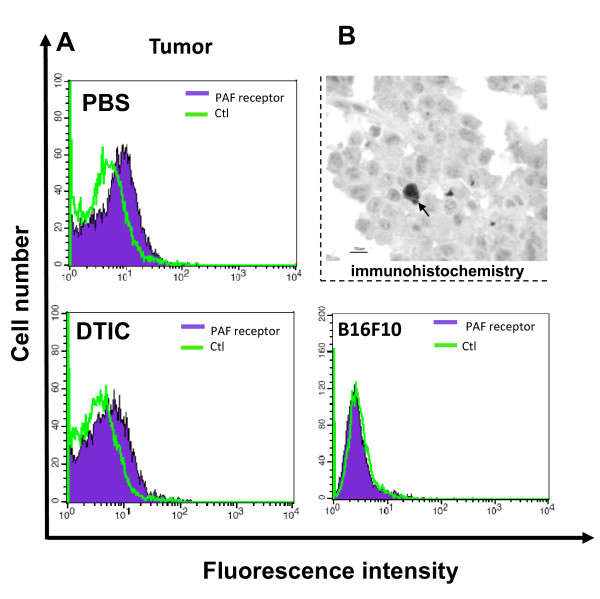
**PAF-R is expressed within the tumour microenvironment**. **(A) **The presence of PAF-R was evaluated by FACS analysis in cell suspensions from B16F10-derived tumours (histograms on the left). Examples from either mock (PBS) or dacarbazine (DTIC)-treated animals are illustrated. The histograms shown represent the negative control (ctl) and reactivity with the antibody to PAF-R. Cells from within the tumours stained positively with the antibody to PAF-R. The histogram on the right represents the absence of reactivity for the antibody to PAF-R in B16F10 cells grown *in vitro*. **(B) **Immunohistochemistry of B16F10-derived tumours using the same antibody to PAF-R, showing specific reactivity to a few cells infiltrating the tumours, consistent with the notion that only a subpopulation of cells within the tumour microenvironment express the PAF-R (arrow indicates a stained cell).

Treatment with WEB2170 alone led to a significant decrease in the frequency of COX-2 positive cells within the microenvironment of melanoma tumours (Fig. 4B). Combination of DTIC with WEB2170 counteracted the reduction in COX-2 positive cells, whose proportion within the tumours was no longer significantly different from that in either the control or DTIC alone groups (Fig. 4B). Microvascular density was significantly lower in both groups receiving WEB2170 (either alone or combined with DTIC), as shown in Fig. 4C. A decrease in microvascular density occurred in parallel with a significant decrease in the relative frequency of galectin-3 positive cells present within the tumour microenvironment, as shown in Fig. 4D. The relative proportion of galectin-3-expressing macrophages/dendritic cells in the splenic white pulp increased significantly upon DTIC treatment (Fig. 4E). Upon combination of both DTIC and WEB2170, the proportion of galectin-3-expressing cells in the white pulp was slightly lower than in the WEB2170 and DTIC treatments alone (PBS and WEB, Fig. 4E). Taken together, these results suggest that cellular elements of the tumour microenvironment are the key targets of the treatment with WEB2170. As shown in Fig. 5, antibodies to PAF-R stained the cells derived from tumours, but did not stain the B16F10 melanoma cells grown *in vitro *(Fig. 5A). At the tissue level, a few cells stained positively with antibodies to PAF-R (Fig. 5B), suggesting that infiltrating mononuclear cells (probably macrophages or dendritic cells) were the major cellular targets for WEB2170 in the model we studied herein.

## Discussion

PAF-R is a G-protein-coupled receptor that can be found in cellular and nuclear membranes. These receptors are coupled to distinct G proteins and can thus trigger distinct signaling pathways. Upon binding of PAF or PAF-like molecules to the PAF-R present in the cell membrane of macrophages, several mediators/cytokines [20] and pro-angiogenic factors such as VEGF are released, angiogenesis being considered one of the important actions of PAF [21,22]. Prostaglandins are also produced by macrophages upon PAF-R engagement and PGE2 exerts anti-inflammatory actions through its interaction with the prostaglandin EP2 and EP4 receptors, which leads to increased intracellular levels of cAMP and inhibition of macrophage activation [6]. These aspects are particularly important when studying tumours, since macrophages that are present in the tumour microenvironment are key actors in both angiogenesis and tumour immune escape. However, the role of PAF/PAF-like molecules and PAF-R as modulators of the tumour microenvironment is still incompletely understood.

We have previously shown that PAF-R-dependent mechanisms are involved in the interaction of macrophages with apoptotic cells [5]. In the present study we examined the effect of apoptotic cells and PAF-R on tumour growth. This was done using two murine tumours: the Ehrlich ascitis tumour and the melanoma B16F10. Apoptotic cells were either inoculated into the tumour or induced by the chemotherapeutic drug DTIC. The role of the PAF-R was evaluated using the antagonist WEB2170. We found that previous inoculation of apoptotic cells into the peritoneal cavity increased EAT growth. The potentiation of tumour growth by apoptotic cells was abolished when animals were treated with WEB2170 before apoptotic cell inoculation. WEB2170 treatment modified the tumour microenvironment since it decreased the levels of VEGF, PGE2 and NO in the ascitis fluid. In the B16F10 melanoma model, treatment with DTIC reduced tumour growth and increased the survival rate only when associated with WEB2170, thus indicating that PAF-R engagement by PAF/PAF-like molecules that are present on apoptotic cells or free in the microenvironment modulate the response of the tumour to chemotherapy. Treatment with WEB2170 alone or associated with DTIC also modified the tumour microenvironment by reducing the number of intra-tumoural blood vessels and tumour infiltration by macrophages/dendritic cells expressing galectin-3, a molecule that is associated with the suppressive M2 phenotype.

These data suggest that PAF-R-dependent mechanisms are able to modify tumour microenvironment elements, including tumour macrophages, during EAT growth. In previous studies in our laboratory, we observed that whereas macrophages taken from the normal peritoneal cavities of mice were able to produce hydrogen peroxide and spread across a glass surface, those taken from mice with EAT retained the round morphology characteristic of non-activated macrophages and were unable to produce hydrogen peroxide [12]. However, macrophage spreading and H_2_O_2 _production in tumour-bearing mice was restored after treatment *in vivo *with antagonists of the PAF-R (BN52021 or SRI63441), and this was accompanied by a significant reduction in EAT growth [11]. This was the first indication that engagement of PAF-R can modify the macrophage phenotype and modulate tumour growth. In the present study we confirmed the EAT growth inhibition by a PAF-R antagonist distinct from that used in previous studies, i.e. WEB2170, which belongs to a group of triazolodiazepinic compounds and has been shown to antagonize the effects of PAF in several cells and tissues [23]. The same dose of WEB2170 that was effective in the latter studies to antagonize the effects of PAF *in vivo *was used in our experiments. Participation of PAF-R was also observed in a solid tumour where WEB2170 significantly reduced the growth of melanoma B16F10.

As tumours grow, a number of tumour and host cells die. The process of cell death is accompanied by oxidation of membrane phospholipids. We have previously shown that apoptotic cells express PAF-like molecules, which share a common or related receptor with oxLDL and PAF in macrophages [5]. We thus evaluated the impact of apoptotic cells on tumour growth and the contribution of WEB2170. It was found that inoculation of apoptotic cells with the EAT cells increased tumour growth and that this effect was abrogated by treatment of mice with WEB2170 prior to the injection of apoptotic cells. In a solid tumour, melanoma B16F10, the combination of WEB2170 with the chemotherapeutic agent DTIC, which induces apoptosis, increased the survival of melanoma-bearing mice. These data suggest that interaction of PAF-like molecules on the surface of apoptotic cells with PAF-R that are present in the tumour microenvironment (presumably in tumour macrophages) has an important modulatory effect during the initial stages after tumour cell implantation. This may also be the case for the B16F10 melanoma model, where the survival time was improved only when DTIC treatment was associated with daily WEB2170 treatment, which began as early as 30 min before melanoma cell inoculation.

Although treatment of melanoma-bearing mice with WEB2170 reduced melanoma growth it did not prolong survival, indicating that the efficacy of treatment was limited to a narrow time window in the initial phase of tumour growth. Another possibility that has to be considered is that increased survival could be related to inhibition of tumour metastasis [14]. Indeed, Im et al [10] demonstrated that PAF increases the metastasis of melanoma B16F10 into the lungs of C57Bl6 mice.

Blocking PAF-R also modified other elements of the tumour microenvironment such as the increased levels of PGE2, NO and VEGF produced during EAT growth. Moreover, in melanoma the PAF-R antagonist reduced the proportion of COX-2-expressing cells, the microvascular density, and the proportion of activated macrophages/dendritic cells expressing galectin-3 within the tumour microenvironment. These alterations did not contribute to the control of the melanoma, unless the animals were given the chemotherapeutic agent DTIC. Interestingly, as shown in Fig. 4, treatment of animals with DTIC alone also failed. A common cause of chemotherapy failure is the phenomenon of tumour cell repopulation [14,24,25]. It is conceivable that release or exposure of PAF/PAF-like molecules by dying cells would affect the phenotype of tumour macrophages and the tumour microenvironment, as suggested by our study. Seo and colleagues [26] showed that exogenously added PAF stimulated B16F10 growth and attenuated etoposide-induced cell death, via activation of anti-apoptotic gene expression. This can be interpreted as showing that the engagement of PAF-R by the agonist produces the same effect as the apoptotic cells in favouring tumour growth by suppressing tumour macrophages. Indeed the frequency of galectin-3, which is a marker of suppressor macrophages, was lower in the group treated with the PAF-R antagonist. Correa et al showed that inoculation of apoptotic cells along with sub-tumourigenic inocula of melanoma cells favoured melanoma growth [8]. This finding strengthens our hypothesis that the presence of apoptotic cells in the tumour microenvironment favours tumour growth.

We have shown the expression of PAF-R in homogenates of melanoma but not in melanoma B16F10 cells kept *in vitro*, which indicates that the receptor is expressed by cells from the tumour microenvironment rather than the tumour cells themselves.

Clearance of dead cells by professional phagocytes such as macrophages may lead to the secretion of cytokines that favour tumour growth [27]. The endogenous lectin galectin-3 is among the activation markers of macrophage/dendritic cells, playing a role as an opsonin [28] and as a modulator of cell migration [29]. Macrophages expressing galectin-3 are driven towards the alternative pathway of activation [30], usually associated with the M2 phenotype, which favours tumour growth and angiogenesis [31]. Here we showed a significant reduction in the proportion of galectin-3-expressing infiltrating macrophages/dendritic cells within the tumour microenvironment of WEB2170-treated mice but not in the spleen white pulp of animals treated with both DTIC and/or WEB2170. Together, these results suggest that engagement of PAF-R interferes with selected pathways in macrophages, changing their phenotype into that of a suppressor. This suggestion is reinforced by the slight but significant decrease in microvascular density observed in WEB2170-treated animals. Indeed, the activation of macrophage PAF-R plays a role in macrophage-derived angiogenesis [31]. As macrophages/dendritic cells are central actors in the maintenance of both normal and tumour tissue homeostasis, novel pharmacological interventions that may control their functions are warranted. For cancer therapy, inhibitors of PAF-R-dependent pathways are promising candidates for adjuvant therapy, as they can act in both tumour cells and host cells, e.g., by attenuating the pro-tumoural activities of suppressor macrophages.

## Conclusions

The data suggest that PAF-R-dependent pathways are activated during experimental tumour growth, modifying the phenotype of tumour macrophages and the microenvironment in such a way as to favour tumour growth. Combination therapy with a PAF-R antagonist and a chemotherapeutic drug may represent a new and promising strategy for the treatment of some tumours.

## Competing interests

The authors declare that they have no competing interests.

## Authors' contributions

SIO carried out the Ehrlich tumour protocols, participated in the melanoma protocols, performed the statistical analysis, participated in the discussion of the results and drafted the manuscript. LNSA designed and carried out the melanoma protocols and participated in the discussion of the results. ACO evaluated the PAF-R expression in melanoma cells. SN carried out the immunohistochemistry. PDF carried out the NO measurements and discussed the NO and PGE2 assay data. MCP participated in the design of the melanoma protocols and discussion of the results. CBSR carried out the experiments on galectin-3 expression. RC and SJ conceived, designed and coordinated the study, discussed the data and critically revised the manuscript. Finally, all the authors read and approved the final manuscript.

## Pre-publication history

The pre-publication history for this paper can be accessed here:

http://www.biomedcentral.com/1471-2407/10/200/prepub
